# (*E*)-6,8-Di­chloro-3-{[(naphthalen-1-ylmeth­yl)iminiumyl]meth­yl}-2*H*-chromen-4-olate

**DOI:** 10.1107/S1600536813018084

**Published:** 2013-07-10

**Authors:** Yoshinobu Ishikawa, Yuya Motohashi

**Affiliations:** a School of Pharmaceutical Sciences, University of Shizuoka, 52-1 Yada, Suruga-ku, Shizuoka 422-8526, Japan

## Abstract

In the title compound, C_21_H_15_Cl_2_NO_2_, the H atom of the –OH group is transferred to the N atom of the imine, forming a zwitterion. Thus, there is formation of a six-membered ring *via* an intra­molecular O⋯H—N, rather than O—H⋯N, hydrogen bond in the mol­ecule. The dihedral angle between the naphthalene ring system and the benzene ring of the 2*H*-chromen system is 87.41 (4)°. In the crystal, the mol­ecules are packed through N—H⋯O, π–π [centroid–centroid distances = 3.744 (3) and 3.780 (3) Å], C—Cl⋯π [Cl⋯centroid = 3.261 (3) Å], C—H⋯π and C—H⋯O inter­actions.

## Related literature

For the biological propertries of similar structures, see: Khan *et al.* (2009 ▶); Tu *et al.* (2013 ▶). For related structures, see: Benaouida *et al.* (2013 ▶); Małecka & Budzisz (2006 ▶).
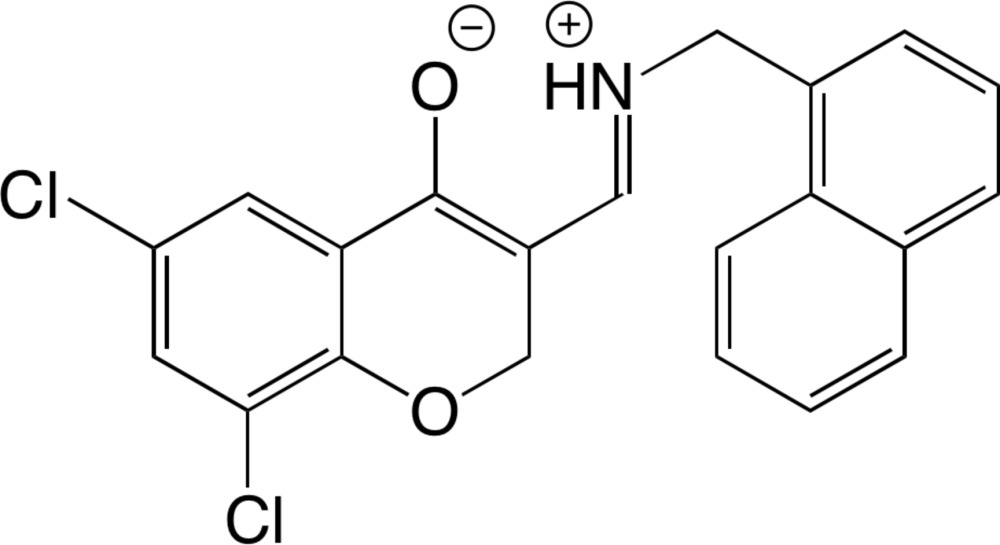


## Experimental

### 

#### Crystal data

C_21_H_15_Cl_2_NO_2_
*M* *_r_* = 384.26
Monoclinic, 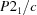

*a* = 16.286 (7) Å
*b* = 8.910 (6) Å
*c* = 12.008 (9) Å
β = 102.65 (4)°
*V* = 1700.2 (19) Å^3^
*Z* = 4
Mo *K*α radiation
μ = 0.40 mm^−1^
*T* = 100 K
0.37 × 0.37 × 0.28 mm

#### Data collection

Rigaku AFC7R diffractometer
4657 measured reflections
3875 independent reflections
3367 reflections with *F* ^2^ > 2.0σ(*F* ^2^)
*R*_int_ = 0.083
3 standard reflections every 150 reflections intensity decay: −0.4%

#### Refinement

*R*[*F* ^2^ > 2σ(*F* ^2^)] = 0.033
*wR*(*F* ^2^) = 0.083
*S* = 1.04
3875 reflections
235 parameters
H-atom parameters constrained
Δρ_max_ = 0.49 e Å^−3^
Δρ_min_ = −0.53 e Å^−3^

### 

Data collection: *WinAFC* (Rigaku, 1999 ▶); cell refinement: *WinAFC*; data reduction: *WinAFC*; program(s) used to solve structure: *SHELXS97* (Sheldrick, 2008 ▶); program(s) used to refine structure: *SHELXL97* (Sheldrick, 2008 ▶); molecular graphics: *CrystalStructure* (Rigaku, 2010 ▶); software used to prepare material for publication: *CrystalStructure*.

## Supplementary Material

Crystal structure: contains datablock(s) General, I. DOI: 10.1107/S1600536813018084/zp2006sup1.cif

Structure factors: contains datablock(s) I. DOI: 10.1107/S1600536813018084/zp2006Isup2.hkl

Additional supplementary materials:  crystallographic information; 3D view; checkCIF report

## Figures and Tables

**Table 1:** Hydrogen-bond geometry (Å, °) *Cg*2 is the centroid of the C4–C9 ring.

*D*—H⋯*A*	*D*—H	H⋯*A*	*D*⋯*A*	*D*—H⋯*A*
N1—H6⋯O2	0.88	2.18	2.794 (2)	126
N1—H6⋯O2^i^	0.88	2.54	3.306 (3)	146
C1—H2*A*⋯O2^ii^	0.99	2.52	3.472 (3)	160
C15—H11⋯*Cg*2^iii^	0.95	2.77	3.682 (3)	160

Symmetry codes: (i) 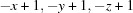; (ii) 
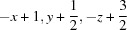
; (iii) 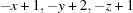.

**Fig. 1.**
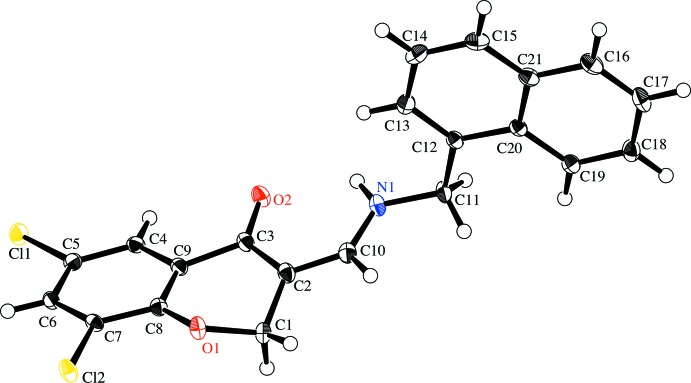
The molecular structure of the title compound, with displacement ellipsoids drawn at the 50% probability level. Hydrogen atoms are shown as small spheres of arbitrary radius.

**Fig. 2.**
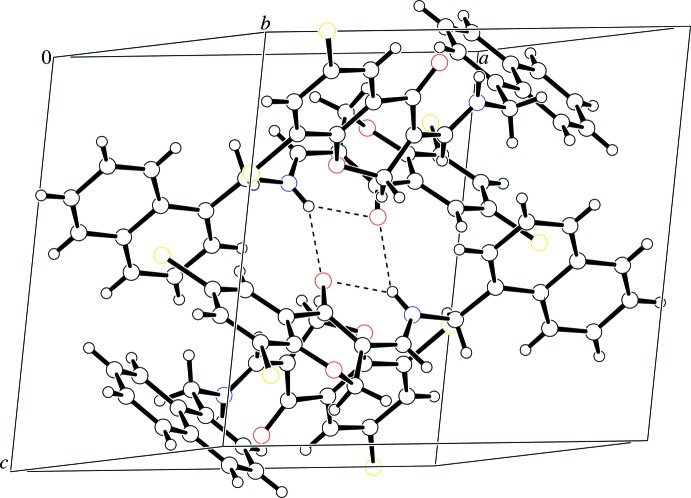
A crystal packing view of the title compound. Intra- and intermolecular N–H···O hydrogen bonds are represented as dashed lines.

## supplementary crystallographic information

### Comment

Schiff bases of 3-formyl chromones have attracted much attention due to their biological functions such as enzyme inhibition (Khan *et al.* 2009; Tu *et al.* 2013). Here we report the crystal structure of the title compound, which was obtained from the condensation reaction of 6,8-dichloro-3-formylchromone with 1-naphthylmethylamine and successive reduction with 2-picoline borane. The structure shows that the H atom of the –OH group is transferred to the N1 atom of the imine, thus forming a zwitterion. As a result, an intramolecular O···H–N [O2···N1 = 2.795 (2) Å], rather than O–H···N, hydrogen bond is formed. The bond distances O2–C3 [1.245 (3) Å], C3–C2 [1.431 (3) Å], C2–C10 [1.377 (3) Å] and C10–N1 [1.329 (3) Å] and torsion angles O2–C3–C2–C10 [3.2 (3)°] and C3–C2–C10–N1 [–2.4 (3)°] in the six-membered ring indicate charge delocalization among the atoms. This effect might be responsible for the preferential reduction of the α,β-unsaturated carbonyl of the synthetic intermediate, rather than reduction of the imine. The dihedral angle between the naphthalene ring and the benzene part of the 2*H*-chromen ring is 87.41 (4)°. In the crystal, the molecules are packed through intermolecular N–H···O, as shown in Figure 2, π···π, C-Cl···π, C-H···π and C-H···O interactions.

### Experimental

1-Naphthylmethylamine (5.0 mmol), 6,8-dichloro-3-formylchromone (5.0 mmol) and 2-picoline borane (5.0 mmol) were dissolved in a mixture of MeOH-AcOH (10:1, 60 ml), and stirred overnight at room temperature. Hydrochloric acid (1 M, 20 ml) was added to the reaction mixture, which was then stirred for 30 min. After neutralization with saturated NaHCO_3_, the mixture was extracted with methylene chloride. The extract was washed with brine, dried over anhydrous Na_2_SO_4_ and purified by column chromatography on silica gel (*n*-hexane: ethyl acetate = 9: 1). The eluted fractions were concentrated and filtered off. Layering *n*-hexane on the filtrate gave single crystals suitable for X-ray diffraction (yield 19%).

### Refinement

The carbon-bound hydrogen atoms were placed in geometrical positions [C–H 0.95 to 0.99 Å, *U*_iso_(H) = 1.2*U*_eq_(C)], and refined using a riding model. The hydrogen atom of the OH group was located near N1 of the imine in a difference Fourier map, and refined with distance constraint [N–H 0.88 Å, *U*_iso_(H) = 1.2*U*_eq_(N)].

### Crystal data

**Table:**

C_21_H_15_Cl_2_NO_2_	*F*(000) = 792.00
*M**_r_* = 384.26	*D*_x_ = 1.501 Mg m^−^^3^
Monoclinic, *P*2_1_/*c*	Mo *K*α radiation, λ = 0.71069 Å
Hall symbol: -P 2ybc	Cell parameters from 25 reflections
*a* = 16.286 (7) Å	θ = 15.7–17.5°
*b* = 8.910 (6) Å	µ = 0.40 mm^−^^1^
*c* = 12.008 (9) Å	*T* = 100 K
β = 102.65 (4)°	Block, yellow
*V* = 1700.2 (19) Å^3^	0.37 × 0.37 × 0.28 mm
*Z* = 4	

### Data collection

**Table:**

Rigaku AFC7R diffractometer	θ_max_ = 27.6°
ω–2θ scans	*h* = −20→21
4657 measured reflections	*k* = 0→11
3875 independent reflections	*l* = −15→8
3367 reflections with *F*^2^ > 2.0σ(*F*^2^)	3 standard reflections every 150 reflections
*R*_int_ = 0.083	intensity decay: −0.4%

### Refinement

**Table:**

Refinement on *F*^2^	Secondary atom site location: difference Fourier map
*R*[*F*^2^ > 2σ(*F*^2^)] = 0.033	Hydrogen site location: inferred from neighbouring sites
*wR*(*F*^2^) = 0.083	H-atom parameters constrained
*S* = 1.04	*w* = 1/[σ^2^(*F*_o_^2^) + (0.0433*P*)^2^ + 0.7479*P*] where *P* = (*F*_o_^2^ + 2*F*_c_^2^)/3
3875 reflections	(Δ/σ)_max_ = 0.001
235 parameters	Δρ_max_ = 0.49 e Å^−^^3^
0 restraints	Δρ_min_ = −0.53 e Å^−^^3^
Primary atom site location: structure-invariant direct methods	

### Special details

**Table:**

Refinement. Refinement was performed using all reflections. The weighted *R*-factor (*wR*) and goodness of fit (*S*) are based on *F*^2^. *R*-factor (gt) are based on *F*. The threshold expression of *F*^2^ > 2.0 σ(*F*^2^) is used only for calculating *R*-factor (gt).

### Fractional atomic coordinates and isotropic or equivalent isotropic displacement parameters (Å^2^)

**Table:**

	*x*	*y*	*z*	*U*_iso_*/*U*_eq_	
Cl1	0.10099 (2)	0.41074 (4)	0.50407 (3)	0.02134 (10)	
Cl2	0.17147 (2)	0.84582 (4)	0.82092 (3)	0.01871 (10)	
O1	0.34501 (6)	0.79385 (11)	0.80754 (9)	0.0176 (2)	
O2	0.43015 (6)	0.52671 (12)	0.57813 (9)	0.0194 (2)	
N1	0.58885 (7)	0.64807 (13)	0.66903 (11)	0.0178 (3)	
C1	0.42933 (8)	0.73258 (16)	0.84317 (12)	0.0173 (3)	
C2	0.46328 (8)	0.67928 (15)	0.74377 (12)	0.0163 (3)	
C3	0.40821 (8)	0.59345 (15)	0.65803 (12)	0.0153 (3)	
C4	0.25901 (8)	0.50521 (16)	0.59487 (11)	0.0149 (3)	
C5	0.17435 (8)	0.52122 (16)	0.59411 (11)	0.0156 (3)	
C6	0.14631 (8)	0.62656 (15)	0.66285 (11)	0.0152 (3)	
C7	0.20515 (8)	0.71380 (15)	0.73498 (11)	0.0141 (3)	
C8	0.29135 (8)	0.69931 (15)	0.73910 (11)	0.0142 (3)	
C9	0.31801 (8)	0.59510 (15)	0.66686 (11)	0.0148 (3)	
C10	0.54680 (8)	0.70260 (15)	0.74329 (12)	0.0166 (3)	
C11	0.67892 (8)	0.67249 (16)	0.68340 (12)	0.0174 (3)	
C12	0.70249 (8)	0.80052 (15)	0.61334 (11)	0.0140 (3)	
C13	0.64295 (9)	0.87459 (16)	0.53406 (12)	0.0176 (3)	
C14	0.66574 (9)	0.99175 (17)	0.46685 (12)	0.0208 (3)	
C15	0.74839 (10)	1.03185 (17)	0.47995 (12)	0.0210 (3)	
C16	0.89800 (9)	0.99932 (17)	0.57563 (13)	0.0212 (3)	
C17	0.95874 (9)	0.93028 (17)	0.65587 (13)	0.0219 (3)	
C18	0.93670 (9)	0.81701 (16)	0.72581 (12)	0.0193 (3)	
C19	0.85400 (8)	0.77372 (15)	0.71310 (12)	0.0157 (3)	
C20	0.78937 (8)	0.84224 (14)	0.63022 (11)	0.0133 (3)	
C21	0.81193 (9)	0.95853 (16)	0.56097 (12)	0.0170 (3)	
H1B	0.4283	0.6476	0.8960	0.0207*	
H2A	0.4672	0.8106	0.8850	0.0207*	
H3	0.2769	0.4334	0.5466	0.0179*	
H4	0.0879	0.6382	0.6603	0.0182*	
H5	0.5777	0.7643	0.8024	0.0199*	
H6	0.5617	0.5962	0.6100	0.0213*	
H7A	0.7048	0.5791	0.6626	0.0209*	
H8B	0.7033	0.6925	0.7651	0.0209*	
H9	0.5855	0.8470	0.5240	0.0211*	
H10	0.6236	1.0424	0.4127	0.0249*	
H11	0.7633	1.1098	0.4341	0.0252*	
H12	0.9135	1.0757	0.5290	0.0255*	
H13	1.0160	0.9588	0.6645	0.0263*	
H14	0.9791	0.7702	0.7820	0.0231*	
H15	0.8400	0.6968	0.7605	0.0188*	

### Atomic displacement parameters (Å^2^)

**Table:**

	*U*^11^	*U*^22^	*U*^33^	*U*^12^	*U*^13^	*U*^23^
Cl1	0.01563 (16)	0.0285 (2)	0.01929 (17)	−0.00243 (13)	0.00266 (12)	−0.00941 (13)
Cl2	0.01504 (16)	0.01710 (17)	0.02549 (18)	−0.00033 (12)	0.00772 (12)	−0.00669 (13)
O1	0.0124 (5)	0.0160 (5)	0.0241 (5)	0.0000 (4)	0.0036 (4)	−0.0044 (4)
O2	0.0161 (5)	0.0212 (5)	0.0225 (5)	0.0025 (4)	0.0078 (4)	−0.0020 (4)
N1	0.0120 (6)	0.0189 (6)	0.0228 (6)	−0.0021 (5)	0.0046 (5)	−0.0003 (5)
C1	0.0116 (6)	0.0192 (7)	0.0204 (7)	0.0005 (5)	0.0020 (5)	−0.0002 (6)
C2	0.0139 (6)	0.0148 (7)	0.0210 (7)	0.0018 (5)	0.0053 (5)	0.0031 (6)
C3	0.0138 (6)	0.0135 (6)	0.0197 (7)	0.0027 (5)	0.0061 (5)	0.0043 (5)
C4	0.0160 (6)	0.0162 (6)	0.0134 (6)	0.0024 (5)	0.0048 (5)	0.0004 (5)
C5	0.0155 (7)	0.0170 (7)	0.0141 (6)	−0.0015 (5)	0.0024 (5)	0.0006 (5)
C6	0.0125 (6)	0.0174 (7)	0.0165 (6)	0.0008 (5)	0.0051 (5)	0.0021 (5)
C7	0.0155 (6)	0.0118 (6)	0.0164 (6)	0.0024 (5)	0.0068 (5)	0.0007 (5)
C8	0.0137 (6)	0.0128 (6)	0.0162 (6)	−0.0001 (5)	0.0039 (5)	0.0022 (5)
C9	0.0138 (6)	0.0150 (7)	0.0165 (6)	0.0018 (5)	0.0053 (5)	0.0033 (5)
C10	0.0146 (6)	0.0142 (6)	0.0206 (7)	0.0000 (5)	0.0032 (5)	0.0037 (6)
C11	0.0110 (6)	0.0173 (7)	0.0245 (7)	0.0012 (5)	0.0050 (5)	0.0044 (6)
C12	0.0140 (6)	0.0132 (6)	0.0155 (6)	0.0001 (5)	0.0051 (5)	−0.0018 (5)
C13	0.0162 (7)	0.0183 (7)	0.0177 (7)	0.0011 (5)	0.0021 (5)	−0.0014 (6)
C14	0.0244 (7)	0.0205 (7)	0.0157 (7)	0.0033 (6)	0.0003 (6)	0.0017 (6)
C15	0.0282 (8)	0.0187 (7)	0.0172 (7)	−0.0015 (6)	0.0072 (6)	0.0027 (6)
C16	0.0231 (8)	0.0187 (7)	0.0246 (7)	−0.0042 (6)	0.0110 (6)	0.0001 (6)
C17	0.0153 (7)	0.0224 (8)	0.0298 (8)	−0.0038 (6)	0.0089 (6)	−0.0056 (6)
C18	0.0150 (7)	0.0206 (7)	0.0219 (7)	0.0030 (6)	0.0035 (6)	−0.0033 (6)
C19	0.0161 (6)	0.0143 (6)	0.0173 (7)	0.0004 (5)	0.0050 (5)	−0.0017 (5)
C20	0.0144 (6)	0.0127 (6)	0.0137 (6)	−0.0000 (5)	0.0050 (5)	−0.0029 (5)
C21	0.0199 (7)	0.0157 (7)	0.0169 (7)	−0.0008 (6)	0.0072 (5)	−0.0021 (6)

### Geometric parameters (Å, º)

**Table:**

Cl1—C5	1.7319 (15)	C15—C21	1.415 (2)
Cl2—C7	1.7313 (17)	C16—C17	1.367 (2)
O1—C1	1.4524 (17)	C16—C21	1.421 (3)
O1—C8	1.3543 (17)	C17—C18	1.409 (3)
O2—C3	1.246 (2)	C18—C19	1.377 (2)
N1—C10	1.330 (3)	C19—C20	1.4185 (19)
N1—C11	1.4554 (19)	C20—C21	1.426 (3)
C1—C2	1.498 (3)	N1—H6	0.880
C2—C3	1.430 (2)	C1—H1B	0.990
C2—C10	1.377 (2)	C1—H2A	0.990
C3—C9	1.496 (2)	C4—H3	0.950
C4—C5	1.384 (2)	C6—H4	0.950
C4—C9	1.3958 (19)	C10—H5	0.950
C5—C6	1.391 (2)	C11—H7A	0.990
C6—C7	1.3817 (19)	C11—H8B	0.990
C7—C8	1.400 (2)	C13—H9	0.950
C8—C9	1.403 (2)	C14—H10	0.950
C11—C12	1.516 (3)	C15—H11	0.950
C12—C13	1.3703 (19)	C16—H12	0.950
C12—C20	1.434 (2)	C17—H13	0.950
C13—C14	1.418 (3)	C18—H14	0.950
C14—C15	1.368 (3)	C19—H15	0.950
			
Cl2···O1	2.9030 (16)	C2···H9^iv^	3.5215
O1···C3	2.878 (3)	C2···H10^vi^	3.2427
O2···N1	2.7938 (19)	C2···H10^iv^	3.5375
O2···C4	2.844 (2)	C3···H2A^ii^	3.3458
O2···C10	2.889 (2)	C3···H5^ii^	2.9718
N1···C3	2.956 (2)	C3···H10^vi^	3.3648
N1···C13	2.846 (3)	C4···H5^ii^	3.4301
C1···C9	2.755 (3)	C4···H7A^iii^	3.3561
C2···C8	2.794 (3)	C4···H8B^ii^	3.2427
C4···C7	2.775 (3)	C4···H11^vi^	3.4591
C5···C8	2.780 (3)	C5···H11^vi^	3.4793
C6···C9	2.800 (3)	C5···H15^ii^	3.4109
C10···C12	3.370 (3)	C6···H11^vi^	3.1293
C10···C13	3.583 (3)	C6···H12^vi^	3.5089
C11···C19	2.937 (3)	C6···H14^viii^	3.5789
C12···C15	2.810 (3)	C7···H11^vi^	2.7047
C13···C21	2.801 (3)	C8···H7A^v^	3.5798
C14···C20	2.819 (3)	C8···H10^vi^	3.4120
C16···C19	2.792 (3)	C8···H11^vi^	2.6837
C17···C20	2.819 (3)	C9···H5^ii^	3.3815
C18···C21	2.806 (3)	C9···H10^vi^	3.5560
Cl1···Cl2^i^	3.4590 (17)	C9···H11^vi^	3.0717
Cl1···C18^ii^	3.531 (3)	C10···H9^iv^	3.3197
Cl1···C18^iii^	3.372 (3)	C10···H10^iv^	3.0585
Cl1···C19^ii^	3.531 (3)	C11···H3^iii^	3.1482
Cl1···C19^iii^	3.298 (3)	C12···H3^iii^	2.9038
Cl1···C20^iii^	3.483 (2)	C13···H1B^v^	2.8981
Cl2···Cl1^iv^	3.4590 (17)	C13···H1B^i^	3.5306
Cl2···C4^iv^	3.544 (3)	C13···H2A^i^	3.4463
Cl2···C5^iv^	3.478 (3)	C13···H3^iii^	3.2759
O1···N1^v^	3.327 (3)	C13···H5^i^	3.0174
O1···C11^v^	3.401 (3)	C14···H1B^v^	2.8448
O2···O2^iii^	3.285 (3)	C14···H5^i^	3.1497
O2···N1^iii^	3.306 (3)	C14···H8B^i^	3.0966
O2···C1^ii^	3.472 (3)	C15···H8B^i^	3.2195
O2···C1^i^	3.542 (3)	C16···H4^v^	3.3653
O2···C10^ii^	3.567 (3)	C16···H13^ix^	3.4908
N1···O1^ii^	3.327 (3)	C16···H14^x^	3.3549
N1···O2^iii^	3.306 (3)	C17···H4^xi^	3.3395
C1···O2^v^	3.472 (3)	C17···H4^v^	3.1019
C1···O2^iv^	3.542 (3)	C17···H12^ix^	3.3585
C4···Cl2^i^	3.544 (3)	C17···H14^x^	3.2288
C5···Cl2^i^	3.478 (3)	C18···H4^xi^	3.1738
C5···C19^ii^	3.302 (3)	C18···H4^v^	3.2351
C6···C16^ii^	3.558 (3)	C18···H13^xii^	3.4745
C6···C17^ii^	3.515 (3)	C20···H3^iii^	3.2717
C6···C18^ii^	3.466 (3)	H1B···C13^ii^	2.8981
C6···C19^ii^	3.479 (3)	H1B···C13^iv^	3.5306
C6···C20^ii^	3.542 (3)	H1B···C14^ii^	2.8448
C6···C21^ii^	3.566 (3)	H1B···H9^ii^	2.8715
C7···C21^ii^	3.399 (3)	H1B···H9^iv^	2.6810
C8···C15^vi^	3.511 (3)	H1B···H10^ii^	2.7785
C10···O2^v^	3.567 (3)	H1B···H10^iv^	3.5696
C10···C13^iv^	3.567 (3)	H2A···O2^v^	2.5248
C10···C14^iv^	3.418 (3)	H2A···O2^iv^	2.9081
C11···O1^ii^	3.401 (3)	H2A···N1^v^	3.1678
C13···C10^i^	3.567 (3)	H2A···N1^iv^	3.5686
C14···C10^i^	3.418 (3)	H2A···C3^v^	3.3458
C15···C8^vi^	3.511 (3)	H2A···C13^iv^	3.4463
C16···C6^v^	3.558 (3)	H2A···H6^v^	2.5906
C17···C6^v^	3.515 (3)	H2A···H6^iv^	2.9221
C18···Cl1^v^	3.531 (3)	H2A···H9^iv^	2.6567
C18···Cl1^iii^	3.372 (3)	H3···Cl2^i^	3.4819
C18···C6^v^	3.466 (3)	H3···C11^iii^	3.1482
C19···Cl1^v^	3.531 (3)	H3···C12^iii^	2.9038
C19···Cl1^iii^	3.298 (3)	H3···C13^iii^	3.2759
C19···C5^v^	3.302 (3)	H3···C20^iii^	3.2717
C19···C6^v^	3.479 (3)	H3···H5^ii^	3.0475
C20···Cl1^iii^	3.483 (2)	H3···H6^iii^	3.5623
C20···C6^v^	3.542 (3)	H3···H7A^iii^	2.5963
C21···C6^v^	3.566 (3)	H3···H8B^ii^	3.0827
C21···C7^v^	3.399 (3)	H3···H9^iii^	3.5808
Cl1···H3	2.8044	H4···Cl1^vii^	3.3003
Cl1···H4	2.8011	H4···C16^ii^	3.3653
Cl2···H4	2.8007	H4···C17^viii^	3.3395
O2···H3	2.5793	H4···C17^ii^	3.1019
O2···H6	2.1816	H4···C18^viii^	3.1738
N1···H9	2.4771	H4···C18^ii^	3.2351
C1···H5	2.5838	H4···H12^vi^	3.4114
C2···H6	2.6121	H4···H13^viii^	3.0915
C3···H1B	2.8456	H4···H13^ii^	3.3779
C3···H2A	3.3086	H4···H14^viii^	2.7925
C3···H3	2.6730	H4···H14^ii^	3.5715
C3···H5	3.2932	H5···O2^v^	2.7607
C3···H6	2.6854	H5···C3^v^	2.9718
C4···H4	3.2775	H5···C4^v^	3.4301
C6···H3	3.2754	H5···C9^v^	3.3815
C8···H1B	2.6266	H5···C13^iv^	3.0174
C8···H2A	3.1711	H5···C14^iv^	3.1497
C8···H3	3.2817	H5···H3^v^	3.0475
C8···H4	3.2853	H5···H9^iv^	2.8161
C9···H1B	2.9757	H5···H10^iv^	3.0576
C10···H1B	2.9791	H6···O1^ii^	3.1444
C10···H2A	2.5432	H6···O2^iii^	2.5402
C10···H7A	3.1414	H6···C1^ii^	3.2859
C10···H8B	2.5054	H6···H2A^ii^	2.5906
C10···H9	3.1166	H6···H2A^i^	2.9221
C11···H5	2.5417	H6···H3^iii^	3.5623
C11···H9	2.6689	H6···H6^iii^	3.4103
C11···H15	2.5932	H7A···Cl2^ii^	2.8708
C12···H5	3.3778	H7A···O1^ii^	2.7154
C12···H6	2.9216	H7A···O2^iii^	3.3604
C12···H10	3.2782	H7A···C4^iii^	3.3561
C12···H15	2.6946	H7A···C8^ii^	3.5798
C13···H6	3.0440	H7A···H3^iii^	2.5963
C13···H7A	3.1064	H8B···C4^v^	3.2427
C13···H8B	3.1775	H8B···C14^iv^	3.0966
C13···H11	3.2717	H8B···C15^iv^	3.2195
C15···H9	3.2633	H8B···H3^v^	3.0827
C15···H12	2.6531	H8B···H10^iv^	3.1963
C16···H11	2.6523	H8B···H11^iv^	3.3862
C16···H14	3.2588	H9···O2^iii^	3.5379
C17···H15	3.2725	H9···C1^i^	3.0434
C18···H12	3.2633	H9···C2^i^	3.5215
C19···H7A	2.9380	H9···C10^i^	3.3197
C19···H8B	2.7588	H9···H1B^v^	2.8715
C19···H13	3.2701	H9···H1B^i^	2.6810
C20···H7A	2.7891	H9···H2A^i^	2.6567
C20···H8B	2.7134	H9···H3^iii^	3.5808
C20···H9	3.2869	H9···H5^i^	2.8161
C20···H11	3.3119	H10···O1^vi^	3.1583
C20···H12	3.3119	H10···N1^i^	3.3222
C20···H14	3.2890	H10···C2^vi^	3.2427
C21···H10	3.2781	H10···C2^i^	3.5375
C21···H13	3.2836	H10···C3^vi^	3.3648
C21···H15	3.3029	H10···C8^vi^	3.4120
H1B···H5	3.0755	H10···C9^vi^	3.5560
H2A···H5	2.2747	H10···C10^i^	3.0585
H5···H6	2.7173	H10···H1B^v^	2.7785
H5···H7A	3.3695	H10···H1B^i^	3.5696
H5···H8B	2.2788	H10···H5^i^	3.0576
H5···H9	3.4527	H10···H8B^i^	3.1963
H6···H7A	2.2832	H11···Cl2^v^	3.5822
H6···H8B	2.7639	H11···Cl2^vi^	3.4771
H6···H9	2.5271	H11···O1^vi^	3.1669
H7A···H9	3.2912	H11···C4^vi^	3.4591
H7A···H15	2.4895	H11···C5^vi^	3.4793
H8B···H9	3.3945	H11···C6^vi^	3.1293
H8B···H15	2.2403	H11···C7^vi^	2.7047
H9···H10	2.3596	H11···C8^vi^	2.6837
H10···H11	2.3125	H11···C9^vi^	3.0717
H11···H12	2.4808	H11···H8B^i^	3.3862
H12···H13	2.3085	H12···Cl2^v^	3.4716
H13···H14	2.3547	H12···C6^vi^	3.5089
H14···H15	2.3174	H12···C17^ix^	3.3585
Cl1···H4^vii^	3.3003	H12···H4^vi^	3.4114
Cl1···H14^ii^	3.3665	H12···H12^ix^	3.3307
Cl1···H15^ii^	3.3698	H12···H13^ix^	2.8245
Cl2···H3^iv^	3.4819	H12···H14^x^	3.0781
Cl2···H7A^v^	2.8708	H13···Cl2^xi^	2.9779
Cl2···H11^ii^	3.5822	H13···C16^ix^	3.4908
Cl2···H11^vi^	3.4771	H13···C18^x^	3.4745
Cl2···H12^ii^	3.4716	H13···H4^xi^	3.0915
Cl2···H13^viii^	2.9779	H13···H4^v^	3.3779
Cl2···H14^viii^	3.1376	H13···H12^ix^	2.8245
Cl2···H15^v^	3.2700	H13···H14^x^	2.8458
O1···H6^v^	3.1444	H13···H15^x^	3.1438
O1···H7A^v^	2.7154	H14···Cl1^v^	3.3665
O1···H10^vi^	3.1583	H14···Cl2^xi^	3.1376
O1···H11^vi^	3.1669	H14···C6^xi^	3.5789
O2···H2A^ii^	2.5248	H14···C16^xii^	3.3549
O2···H2A^i^	2.9081	H14···C17^xii^	3.2288
O2···H5^ii^	2.7607	H14···H4^xi^	2.7925
O2···H6^iii^	2.5402	H14···H4^v^	3.5715
O2···H7A^iii^	3.3604	H14···H12^xii^	3.0781
O2···H9^iii^	3.5379	H14···H13^xii^	2.8458
N1···H2A^ii^	3.1678	H15···Cl1^v^	3.3698
N1···H2A^i^	3.5686	H15···Cl2^ii^	3.2700
N1···H10^iv^	3.3222	H15···C5^v^	3.4109
C1···H6^v^	3.2859	H15···H13^xii^	3.1438
C1···H9^iv^	3.0434		
			
C1—O1—C8	112.82 (12)	C19—C20—C21	118.39 (13)
C10—N1—C11	121.31 (12)	C15—C21—C16	121.33 (15)
O1—C1—C2	112.00 (12)	C15—C21—C20	119.55 (14)
C1—C2—C3	117.41 (13)	C16—C21—C20	119.12 (13)
C1—C2—C10	119.51 (12)	C10—N1—H6	119.342
C3—C2—C10	122.82 (14)	C11—N1—H6	119.346
O2—C3—C2	124.70 (13)	O1—C1—H1B	109.210
O2—C3—C9	120.73 (12)	O1—C1—H2A	109.215
C2—C3—C9	114.49 (13)	C2—C1—H1B	109.214
C5—C4—C9	119.64 (14)	C2—C1—H2A	109.212
Cl1—C5—C4	119.70 (12)	H1B—C1—H2A	107.905
Cl1—C5—C6	118.85 (11)	C5—C4—H3	120.177
C4—C5—C6	121.43 (12)	C9—C4—H3	120.180
C5—C6—C7	118.60 (13)	C5—C6—H4	120.699
Cl2—C7—C6	119.28 (11)	C7—C6—H4	120.701
Cl2—C7—C8	119.20 (10)	N1—C10—H5	116.499
C6—C7—C8	121.52 (13)	C2—C10—H5	116.493
O1—C8—C7	118.23 (13)	N1—C11—H7A	108.592
O1—C8—C9	122.75 (13)	N1—C11—H8B	108.590
C7—C8—C9	118.88 (12)	C12—C11—H7A	108.590
C3—C9—C4	120.27 (13)	C12—C11—H8B	108.589
C3—C9—C8	119.48 (12)	H7A—C11—H8B	107.556
C4—C9—C8	119.90 (13)	C12—C13—H9	119.454
N1—C10—C2	127.01 (13)	C14—C13—H9	119.453
N1—C11—C12	114.70 (11)	C13—C14—H10	119.980
C11—C12—C13	121.42 (13)	C15—C14—H10	119.986
C11—C12—C20	118.60 (11)	C14—C15—H11	119.628
C13—C12—C20	119.98 (14)	C21—C15—H11	119.617
C12—C13—C14	121.09 (14)	C17—C16—H12	119.532
C13—C14—C15	120.03 (13)	C21—C16—H12	119.524
C14—C15—C21	120.75 (15)	C16—C17—H13	119.901
C17—C16—C21	120.94 (15)	C18—C17—H13	119.892
C16—C17—C18	120.21 (14)	C17—C18—H14	119.822
C17—C18—C19	120.35 (13)	C19—C18—H14	119.826
C18—C19—C20	120.98 (14)	C18—C19—H15	119.511
C12—C20—C19	123.04 (13)	C20—C19—H15	119.512
C12—C20—C21	118.57 (12)		
			
C1—O1—C8—C7	155.30 (11)	O1—C8—C9—C4	−177.29 (11)
C1—O1—C8—C9	−29.15 (17)	C7—C8—C9—C3	171.33 (11)
C8—O1—C1—C2	52.17 (15)	C7—C8—C9—C4	−1.77 (19)
C8—O1—C1—H1B	−68.9	N1—C11—C12—C13	−6.86 (19)
C8—O1—C1—H2A	173.3	N1—C11—C12—C20	174.05 (11)
C10—N1—C11—C12	−98.17 (16)	H7A—C11—C12—C13	114.8
C10—N1—C11—H7A	140.2	H7A—C11—C12—C20	−64.3
C10—N1—C11—H8B	23.5	H8B—C11—C12—C13	−128.5
C11—N1—C10—C2	−175.34 (12)	H8B—C11—C12—C20	52.4
C11—N1—C10—H5	4.7	C11—C12—C13—C14	−178.41 (12)
H6—N1—C10—C2	4.7	C11—C12—C13—H9	1.6
H6—N1—C10—H5	−175.3	C11—C12—C20—C19	−2.90 (19)
H6—N1—C11—C12	81.8	C11—C12—C20—C21	177.44 (11)
H6—N1—C11—H7A	−39.8	C13—C12—C20—C19	178.00 (12)
H6—N1—C11—H8B	−156.5	C13—C12—C20—C21	−1.66 (19)
O1—C1—C2—C3	−44.51 (16)	C20—C12—C13—C14	0.7 (2)
O1—C1—C2—C10	141.16 (11)	C20—C12—C13—H9	−179.3
H1B—C1—C2—C3	76.6	C12—C13—C14—C15	0.5 (3)
H1B—C1—C2—C10	−97.7	C12—C13—C14—H10	−179.5
H2A—C1—C2—C3	−165.6	H9—C13—C14—C15	−179.5
H2A—C1—C2—C10	20.1	H9—C13—C14—H10	0.5
C1—C2—C3—O2	−171.00 (12)	C13—C14—C15—C21	−0.6 (3)
C1—C2—C3—C9	12.11 (17)	C13—C14—C15—H11	179.4
C1—C2—C10—N1	171.75 (12)	H10—C14—C15—C21	179.4
C1—C2—C10—H5	−8.3	H10—C14—C15—H11	−0.6
C3—C2—C10—N1	−2.3 (3)	C14—C15—C21—C16	−179.85 (13)
C3—C2—C10—H5	177.7	C14—C15—C21—C20	−0.4 (3)
C10—C2—C3—O2	3.1 (3)	H11—C15—C21—C16	0.1
C10—C2—C3—C9	−173.76 (12)	H11—C15—C21—C20	179.6
O2—C3—C9—C4	8.74 (19)	C17—C16—C21—C15	178.49 (13)
O2—C3—C9—C8	−164.33 (12)	C17—C16—C21—C20	−0.9 (3)
C2—C3—C9—C4	−174.23 (11)	C21—C16—C17—C18	−0.1 (3)
C2—C3—C9—C8	12.69 (17)	C21—C16—C17—H13	179.9
C5—C4—C9—C3	−172.09 (11)	H12—C16—C17—C18	179.9
C5—C4—C9—C8	1.0 (2)	H12—C16—C17—H13	−0.1
C9—C4—C5—Cl1	179.53 (11)	H12—C16—C21—C15	−1.5
C9—C4—C5—C6	0.7 (2)	H12—C16—C21—C20	179.1
H3—C4—C5—Cl1	−0.5	C16—C17—C18—C19	0.7 (3)
H3—C4—C5—C6	−179.3	C16—C17—C18—H14	−179.3
H3—C4—C9—C3	7.9	H13—C17—C18—C19	−179.3
H3—C4—C9—C8	−179.0	H13—C17—C18—H14	0.7
Cl1—C5—C6—C7	179.64 (9)	C17—C18—C19—C20	−0.3 (3)
Cl1—C5—C6—H4	−0.4	C17—C18—C19—H15	179.7
C4—C5—C6—C7	−1.6 (2)	H14—C18—C19—C20	179.7
C4—C5—C6—H4	178.4	H14—C18—C19—H15	−0.3
C5—C6—C7—Cl2	179.84 (11)	C18—C19—C20—C12	179.65 (12)
C5—C6—C7—C8	0.70 (19)	C18—C19—C20—C21	−0.7 (2)
H4—C6—C7—Cl2	−0.2	H15—C19—C20—C12	−0.3
H4—C6—C7—C8	−179.3	H15—C19—C20—C21	179.3
Cl2—C7—C8—O1	−2.47 (17)	C12—C20—C21—C15	1.54 (19)
Cl2—C7—C8—C9	−178.20 (8)	C12—C20—C21—C16	−179.02 (11)
C6—C7—C8—O1	176.67 (12)	C19—C20—C21—C15	−178.13 (12)
C6—C7—C8—C9	0.94 (19)	C19—C20—C21—C16	1.30 (19)
O1—C8—C9—C3	−4.19 (19)		

Symmetry codes: (i) *x*, −*y*+3/2, *z*−1/2; (ii) −*x*+1, *y*−1/2, −*z*+3/2; (iii) −*x*+1, −*y*+1, −*z*+1; (iv) *x*, −*y*+3/2, *z*+1/2; (v) −*x*+1, *y*+1/2, −*z*+3/2; (vi) −*x*+1, −*y*+2, −*z*+1; (vii) −*x*, −*y*+1, −*z*+1; (viii) *x*−1, *y*, *z*; (ix) −*x*+2, −*y*+2, −*z*+1; (x) −*x*+2, *y*+1/2, −*z*+3/2; (xi) *x*+1, *y*, *z*; (xii) −*x*+2, *y*−1/2, −*z*+3/2.

### Hydrogen-bond geometry (Å, º)

Cg2 is the centroid of the C4–C9 ring.

**Table:**

*D*—H···*A*	*D*—H	H···*A*	*D*···*A*	*D*—H···*A*
N1—H6···O2	0.88	2.18	2.794 (2)	126
N1—H6···O2^iii^	0.88	2.54	3.306 (3)	146
C1—H2*A*···O2^v^	0.99	2.52	3.472 (3)	160
C15—H11···*Cg*2^vi^	0.95	2.77	3.682 (3)	160

Symmetry codes: (iii) −*x*+1, −*y*+1, −*z*+1; (v) −*x*+1, *y*+1/2, −*z*+3/2; (vi) −*x*+1, −*y*+2, −*z*+1.
